# Robotic bronchoscopy in early lung cancer: a biomarker-driven platform for genomic profiling and immunotherapy-oriented decision-making

**DOI:** 10.3389/fgene.2026.1980607

**Published:** 2026-10-27

**Authors:** Dengxia Zhang, Yahui Han, Haonan Zhang, Chaoyang Yu, Shiqian Zhang, Teng Li

**Affiliations:** 1 Zhoukou Vocational and Technical College, Zhoukou, Henan, China; 2 School of Pharmacy, Harbin Medical University School of Pharmacy, Harbin, China; 3 Department of Head and Neck Surgical Oncology, National Cancer Center/National Clinical Research Center for Cancer/Cancer Hospital, Chinese Academy of Medical Sciences and Peking Union Medical College, Beijing, China; 4 Department of Vascular Surgery, The First Affiliated Hospital of Tsinghua University, Beijing, China; 5 School of Medicine, Nankai University, Tianjin, China; 6 Department of Colorectal Surgery, Tianjin Union Medical Center and the First Affiliated Hospital of Nankai University, Tianjin, China; 7 Department of Ophthalmology, The First Affiliated Hospital of Zhengzhou University, Zhengzhou University, Zhengzhou, Henan, China

**Keywords:** early lung cancer, genomic profiling, immune biomarkers, molecular biomarkers, precision oncology, robotic bronchoscopy

## Abstract

Robotic bronchoscopy is increasingly used to sample peripheral pulmonary lesions as lung cancer screening detects more small, distal, subsolid and ground-glass nodules. Most evidence has assessed the technology by diagnostic yield and safety, but these endpoints only partly capture clinical value. In early lung cancer care, the relevant question is whether bronchoscopy provides information that is sufficient for diagnosis, staging, molecular profiling and treatment-ready decision-making. This state-of-the-art review examines robotic bronchoscopy as a potential diagnosis-to-treatment platform rather than an isolated navigation device. We summarise evidence on distal airway access, procedural stability, CT-to-body divergence, tool-in-lesion confirmation, cone-beam CT, mobile cone-beam CT and radial endobronchial ultrasound. We also discuss tissue stewardship, rapid on-site evaluation, cell block preparation, next-generation sequencing, PD-L1 testing and same-session endobronchial ultrasound-guided transbronchial needle aspiration (EBUS-TBNA) and, when appropriate, endoscopic ultrasound with bronchoscope-guided fine-needle aspiration (EUS-B-FNA) as determinants of molecular adequacy and clinical actionability. We propose clinically actionable yield as a pragmatic, complementary, and currently unvalidated reporting construct linking lesion access, confirmed targeting, pathological diagnosis, molecular adequacy, staging completeness, and management decisions. For prospective evaluation, Level 5 (treatment decision informed or changed) should be assessed within 30 days after the index bronchoscopic procedure or before definitive therapy, whichever occurs first. Bronchoscopic ablation is considered as a future therapeutic extension, but current evidence remains early and does not establish equivalence with surgery, stereotactic body radiotherapy or percutaneous ablation. Future studies should evaluate decision-centred and patient-centred endpoints, including molecular-testing success, repeat biopsy avoidance, time to treatment, local control, pulmonary function, quality of life and cost-effectiveness.

## 1 Introduction

The expanding use of low-dose computed tomography screening has shifted the clinical landscape of lung cancer detection. Increasing numbers of small, peripheral, subsolid, and ground-glass pulmonary lesions are now identified before symptoms develop and before radiological progression becomes obvious (Potter et al., 2022; Christensen et al., 2024). This earlier detection creates a diagnostic paradox. Although clinicians are finding more potentially curable lesions, they are also being asked to obtain reliable pathological and molecular information from lesions that are smaller, more distal, less solid, and often technically more difficult to sample (Zhong et al., 2024; Fernandez-Bussy et al., 2024a).

Conventional diagnostic pathways have not fully adapted to this change. CT-guided transthoracic needle biopsy can provide high diagnostic accuracy for selected peripheral lesions, but it carries relevant risks, particularly pneumothorax, bleeding, chest pain, and procedure-related morbidity in patients with emphysema or limited pulmonary reserve (Lentz et al., 2025; Huo et al., 2020). Conventional flexible bronchoscopy is generally safer but has historically provided lower diagnostic yield for small peripheral pulmonary lesions, especially when lesions are located beyond the reach of standard bronchoscopic tools or lack a favourable bronchus sign (Kim et al., 2023; Nadig et al., 2023; Balasubramanian et al., 2024). Electromagnetic navigation bronchoscopy, radial endobronchial ultrasound, ultrathin bronchoscopy, and virtual bronchoscopic navigation have improved access to peripheral lesions, but each remains limited by respiratory motion, airway deformation, atelectasis, CT-to-body divergence, and uncertainty regarding whether the biopsy tool is truly within the target lesion (Senyei et al., 2024).

Robotic bronchoscopy has emerged in response to these limitations. By improving catheter stability, distal airway access, and controlled tool delivery, robotic systems offer a more precise endobronchial route to peripheral pulmonary lesions (Paez et al., 2025a; Murgu et al., 2025). Recent prospective and randomized evidence suggests that robotic bronchoscopy has moved beyond early feasibility studies and is becoming part of contemporary diagnostic practice for peripheral pulmonary lesions. However, the clinical value of robotic bronchoscopy is not best judged by the robot alone. Its performance depends on the wider procedural ecosystem, including real-time imaging, cone-beam CT or mobile cone-beam CT, radial endobronchial ultrasound, fluoroscopic confirmation, rapid on-site evaluation, tissue handling, molecular pathology, and same-session mediastinal staging (Husta et al., 2026; Fernande et al., 2026).

Most studies of robotic bronchoscopy have focused on diagnostic yield and procedural safety. These endpoints are necessary, but they are no longer sufficient. In early lung cancer care, the central clinical question is not only whether a bronchoscopic procedure can reach and diagnose a peripheral lesion, but whether it can generate enough actionable information to guide the next step in care. A malignant diagnosis without adequate tissue for clinically indicated biomarker testing—including next-generation sequencing and PD-L1 assessment when required—or without appropriate staging may still leave the multidisciplinary team with an incomplete decision pathway (Lee-Mateus et al., 2024; Chaddha et al., 2025; Miller et al., 2025). Conversely, a bronchoscopic procedure that provides diagnosis, staging information, molecular adequacy, and treatment direction can reduce repeated procedures and accelerate definitive management (Fernandez-Bussy et al., 2025a). This perspective is increasingly relevant in biomarker-driven lung cancer care. The clinical value of a bronchoscopic specimen is determined not only by histological diagnosis but also by its capacity to support genomic and immune biomarker assessment. Genomic profiling can identify molecular alterations that influence targeted-treatment strategies and trial eligibility, whereas immune biomarkers such as PD-L1 may contribute to immunotherapy-oriented treatment planning. As biomarker-guided systemic therapy moves into earlier stages of non-small cell lung cancer, preservation of adequate tissue for both genomic and immunological characterization becomes an increasingly important component of the diagnostic pathway.

This review therefore examines robotic bronchoscopy not simply as a navigation technology, but as a potential diagnosis-to-treatment platform for peripheral pulmonary lesions. We first define the diagnostic gap created by modern lung cancer screening and small-lesion detection. We then discuss how robotic bronchoscopy, real-time imaging, and CT-to-body divergence correction can improve lesion access and confirmation. We next examine tissue adequacy, molecular profiling, same-session staging, and precision biopsy as essential components of clinical value. Finally, we appraise the emerging therapeutic extension of robotic bronchoscopy, including bronchoscopic ablation, while emphasising the current limitations of evidence and the need for future trials to move beyond diagnostic yield toward clinically actionable yield (Figure 1).

**FIGURE 1:**
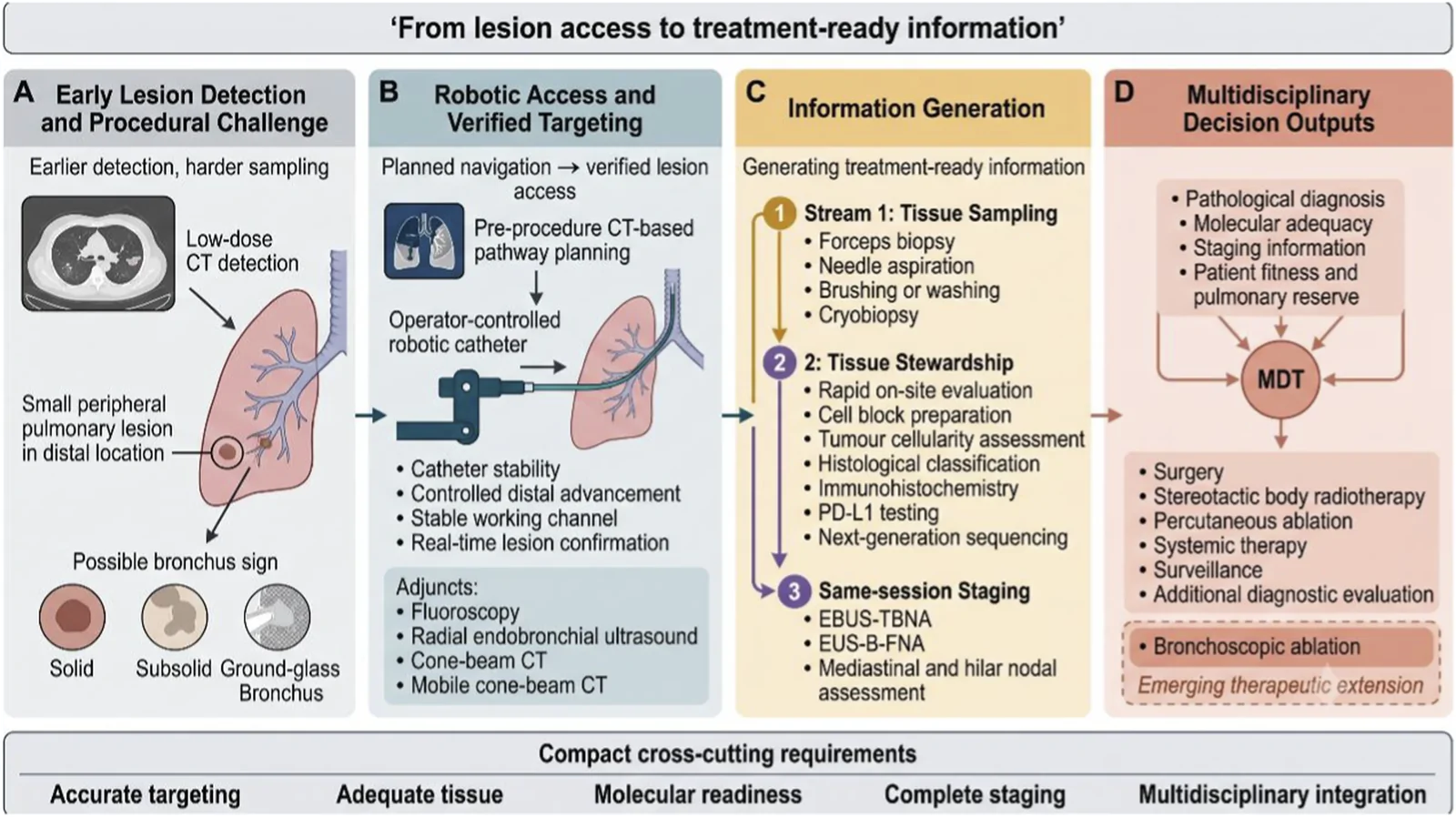
Robotic bronchoscopy as a diagnosis-to-treatment platform in early lung cancer care. **(A)** Earlier detection of small peripheral pulmonary lesions creates increasing procedural challenges. **(B)** Robotic bronchoscopy provides stable distal access, while adjunct imaging enables transition from planned navigation to verified lesion targeting. **(C)** Tissue acquisition, tissue stewardship and same-session nodal staging generate treatment-ready information. **(D)** Integration of pathological, molecular and staging information supports multidisciplinary treatment decisions, with bronchoscopic ablation representing an emerging therapeutic extension.

## 2 The unresolved diagnostic gap in peripheral pulmonary lesions

The diagnostic challenge of peripheral pulmonary lesions is often framed as a problem of access. This framing is incomplete. Although reaching a small distal lesion is a prerequisite for diagnosis, clinically useful bronchoscopy requires a sequence of successful steps: identifying the correct target, reaching the planned bronchoscopic region, confirming that the biopsy tool is within the lesion, obtaining adequate tissue, and translating the obtained material into a treatment decision (Gonzalez et al., 2024; Leonard et al., 2024). Failure at any step may convert an apparently successful procedure into an incomplete diagnostic pathway (Nadig et al., 2023; Balasubramanian et al., 2024).

The first barrier is anatomical and radiological. Peripheral pulmonary lesions detected through screening are frequently small, distal, subsolid, or ground-glass dominant (Gomes et al., 2025; Abia-Trujillo et al., 2023). These features reduce the reliability of conventional bronchoscopic sampling because the lesion may be located beyond the effective reach of standard bronchoscopic tools or may not produce a clear endobronchial target. The absence of a bronchus sign further reduces the likelihood that a planned airway route will lead directly to the lesion (Ali et al., 2018). Even when a bronchus sign is present, airway tortuosity and distal branching may limit the ability to maintain a stable tool position during sampling (Ali et al., 2018; Kalchiem-Dekel et al., 2022).

The second barrier is dynamic. Peripheral lesions are not static targets. The procedural lung differs from the pre-procedure CT lung because of anaesthesia, ventilation strategy, atelectasis, respiratory motion, patient positioning, airway deformation, and mechanical displacement caused by instruments (Sagar et al., 2020; Salahuddin et al., 2022). This mismatch, commonly described as CT-to-body divergence, can shift the actual lesion away from the planned navigation target (Bhadra et al., 2023a). As a result, a bronchoscope may appear to have reached the expected virtual location while the biopsy tool remains adjacent to, rather than within, the lesion (Ho et al., 2025).

The third barrier is procedural confirmation. Traditional fluoroscopy provides limited three-dimensional information, and radial endobronchial ultrasound improves lesion localisation but remains operator-dependent and may not fully resolve whether the sampling tool is optimally positioned (Moulton et al., 2024). Cone-beam CT and mobile cone-beam CT have therefore become increasingly important adjuncts because they can provide intra-procedural confirmation, identify CT-to-body divergence, and allow adjustment of the biopsy trajectory (Husta et al., 2024; Sridharan et al., 2022). These technologies shift the procedural endpoint from planned navigation to verified lesion access (Li et al., 2025).

The fourth barrier is tissue adequacy. A pathological diagnosis alone may no longer be sufficient in suspected lung cancer. Contemporary thoracic oncology increasingly requires tissue that can support histological classification, immunohistochemistry, PD-L1 testing, and next-generation sequencing (Connolly et al., 2023; Kerr et al., 2024). Small or crushed samples, excessive blood contamination, low tumour cellularity, and inadequate cell block preparation may all compromise downstream testing (Oberg et al., 2022). A bronchoscopic procedure that confirms malignancy but fails to provide adequate material for molecular profiling may still require repeat biopsy, delaying treatment and increasing procedural burden (Roy-Chowdhuri et al., 2020).

The fifth barrier is clinical integration. Peripheral pulmonary lesions are managed through multidisciplinary decisions that may include surgery, stereotactic body radiotherapy, percutaneous ablation, bronchoscopic ablation, systemic therapy, or surveillance (Howington et al., 2025; Panel et al., 2023). The value of a diagnostic procedure therefore depends on how efficiently it informs that pathway. Same-session mediastinal staging, tissue triage, rapid on-site evaluation, molecular testing readiness, and communication with pathology and oncology teams determine whether bronchoscopic diagnosis becomes actionable clinical information (Ter Woerds et al., 2025; Chen et al., 2025).

For these reasons, the diagnostic gap in peripheral pulmonary lesions is best understood as more than low diagnostic yield. It is a gap between lesion detection and treatment-ready information. Robotic bronchoscopy is best positioned to address this gap when it is integrated with real-time imaging, tool-in-lesion confirmation, tissue stewardship, and staging, rather than when it is used as an isolated navigation device.

## 3 Robotic bronchoscopy: Stability, reach, and procedural control

Robotic bronchoscopy was developed to improve endobronchial access to peripheral pulmonary lesions by addressing several limitations of conventional and navigation-assisted bronchoscopy. Its main procedural advantages are not simply automation or novelty, but improved catheter stability, controlled distal advancement, enhanced tool alignment, and the ability to maintain position during repeated sampling (Chen and Gillespie, 2018; Yarmus et al., 2020). These features are particularly relevant for small peripheral lesions, where minor displacement of the bronchoscope or biopsy tool can determine whether the sample is diagnostic (Thiboutot et al., 2022; Saghaie et al., 2024).

The value of robotic bronchoscopy can be interpreted through its procedural functions. First, robotic platforms may improve reach by allowing the operator to advance a steerable catheter into more distal airways while maintaining a stable working channel (Fielding et al., 2019; Chen et al., 2021). Second, they may improve control by reducing the instability and recoil that can occur when tools are passed through a flexible bronchoscope. Third, robotic platforms may support more reproducible repeated tool deployment by maintaining catheter position during sequential sampling (Husta et al., 2026; Fernande et al., 2026). These functions are central to procedural reliability, especially when tissue quantity and quality are required for molecular testing (Reisenauer et al., 2022; Meng et al., 2023).

Clinical evidence for robotic bronchoscopy has matured rapidly. Early studies mainly established feasibility and safety, whereas more recent evidence includes prospective multicentre studies, comparative cohort data, large real-world cohorts, and meta-analyses (Paez et al., 2025a; Xie et al., 2024). This evolution is important because it moves robotic bronchoscopy beyond the stage of technical enthusiasm and into a phase where its value can be tested against existing diagnostic pathways. However, the interpretation of these studies remains complicated by heterogeneous definitions of diagnostic yield, differences in lesion size and morphology, variation in imaging adjuncts, and differences in follow-up confirmation of benign results comparative cohort data (Khan et al., 2023; Ali et al., 2023).

Robotic bronchoscopy is not best evaluated as a stand-alone technology. Its performance is strongly influenced by the imaging and sampling ecosystem in which it is used. A robotic catheter can provide stable access, but stability does not guarantee that the tool is within the lesion. Real-time imaging, cone-beam CT, mobile cone-beam CT, radial endobronchial ultrasound, and fluoroscopic confirmation are therefore essential for converting planned navigation into verified lesion access (Kalchiem Dekel et al., 2021; Styrvoky et al., 2022; Chan et al., 2026). Similarly, diagnostic success depends not only on reaching the lesion, but on selecting appropriate sampling tools, processing tissue correctly, and ensuring adequacy for molecular analysis.

This distinction has practical implications. A centre using robotic bronchoscopy without real-time three-dimensional imaging, rapid feedback on sample adequacy, or a coordinated tissue stewardship pathway may obtain different results from a centre using the same robotic platform within a mature procedural ecosystem (Bott et al., 2025). Differences between expert centres and general practice can therefore be expected (Brock et al., 2025). Future studies need to report not only the robotic platform used, but also imaging adjuncts, ventilation strategy, anaesthesia approach, lesion confirmation methods, sampling tools, number of passes, pathology processing, and molecular-testing success (Brock et al., 2026; Funes-Ferrada et al., 2025).

The next phase of robotic bronchoscopy will be defined by whether it can improve clinical pathways rather than procedural metrics alone. Stable distal access is necessary, but it is only the first step. The broader goal is to combine robotic control with real-time confirmation, adequate tissue acquisition, same-session staging, and multidisciplinary treatment planning. In this sense, robotic bronchoscopy is best viewed less as a device and more as a procedural architecture for early lung cancer care.

## 4 CT-to-body divergence and real-time lesion confirmation

CT-to-body divergence is one of the central technical barriers in robotic bronchoscopy for peripheral pulmonary lesions. Robotic navigation is typically planned using pre-procedure CT images, but the lung encountered during the procedure is not identical to the lung represented in the planning scan. Anaesthesia, positive-pressure ventilation, atelectasis, patient positioning, respiratory motion, airway deformation, and mechanical displacement by bronchoscopic tools can all alter the spatial relationship between the airway and the target lesion (Pritchett et al., 2020a; Keshishyan et al., 2025). As a result, the planned virtual target and the actual intra-procedural lesion may no longer coincide (Vo et al., 2026; Bruinen et al., 2025).

This mismatch is clinically important because robotic stability does not guarantee biological accuracy. A robotic catheter may remain stable at the planned target location while the lesion itself has shifted away from that location (Pritchett et al., 2020b; Mahajan et al., 2026). In such cases, navigation success may be misleading if it is not accompanied by lesion confirmation. The practical question is therefore not only whether the bronchoscope followed the planned pathway, but whether the biopsy tool is actually inside the lesion at the time of sampling (Bhadra et al., 2023b).

Traditional fluoroscopy provides useful procedural guidance, but its two-dimensional nature limits accurate confirmation of small, peripheral, or subsolid lesions. Radial endobronchial ultrasound can improve lesion localisation and provides important information about whether the probe is concentric, eccentric, or adjacent to the lesion (Moulton et al., 2024; Shaller et al., 2024). However, radial endobronchial ultrasound remains operator-dependent and may not fully resolve the three-dimensional relationship between the sampling tool and the target. These limitations have increased interest in cone-beam CT and mobile cone-beam CT as adjuncts to robotic bronchoscopy (Cumbo-Nacheli et al., 2022).

Real-time three-dimensional imaging changes the procedural logic of robotic bronchoscopy (Bash et al., 2024; Benn et al., 2026). Instead of relying exclusively on pre-procedure planning and virtual navigation, cone-beam CT or mobile cone-beam CT allows the operator to verify the actual lesion position, identify CT-to-body divergence, adjust the catheter or tool trajectory, and document tool-in-lesion status before tissue acquisition. This turns lesion confirmation into a measurable procedural endpoint rather than an assumed consequence of navigation (Benn et al., 2026).

Tool-in-lesion confirmation can therefore be treated as an intermediate endpoint between lesion access and diagnostic success. It is conceptually distinct from both navigation completion and pathological diagnosis. Navigation completion indicates that the robotic system has reached the planned target region. Tool-in-lesion confirmation indicates that the sampling device is positioned within the actual lesion. Pathological diagnosis then depends on whether the sampled tissue contains representative and adequate material. Separating these steps can help identify where a procedure fails and why diagnostic yield varies between studies and centres (Zhong et al., 2024; Huo et al., 2020).

This distinction is especially relevant for small nodules, ground-glass lesions, and subsolid lesions. These targets are more susceptible to sampling error because they may be difficult to visualise fluoroscopically, may contain low tumour cellularity, and may be displaced by atelectasis or ventilation-related changes (Zhang et al., 2026; Huang et al., 2024). In such lesions, a non-diagnostic result may reflect failure of access, failure of confirmation, inadequate sampling, or true benign disease. Without intra-procedural confirmation and standardised follow-up, these possibilities are difficult to distinguish (Ost et al., 2026).

Future studies of robotic bronchoscopy therefore need to report CT-to-body divergence and lesion confirmation more systematically. Important variables include lesion size, lesion density, bronchus sign, distance from pleura, airway generation, anaesthesia and ventilation strategy, presence of atelectasis, use of cone-beam CT or mobile cone-beam CT, radial endobronchial ultrasound view, tool-in-lesion status, and whether the sampling trajectory was modified after intra-procedural imaging. Reporting these variables would make it possible to compare not only diagnostic yield, but the technical reliability of the pathway leading to diagnosis (Xie et al., 2025; Bruinen et al., 2026).

The broader implication is that real-time lesion confirmation is not merely a technical enhancement. It is a prerequisite for evaluating robotic bronchoscopy as a diagnosis-to-treatment platform. If future applications include bronchoscopic ablation, local drug delivery, or same-session diagnosis and therapy, the tolerance for targeting error will be much lower than in diagnostic biopsy alone (Xu et al., 2025; Xu et al., 2026). For therapeutic extension, confirming that the tool is within the lesion becomes not only diagnostically relevant, but oncologically essential (Figure 2) (Zhang et al., 2025; Fernandez-Bussy et al., 2026).

**FIGURE 2:**
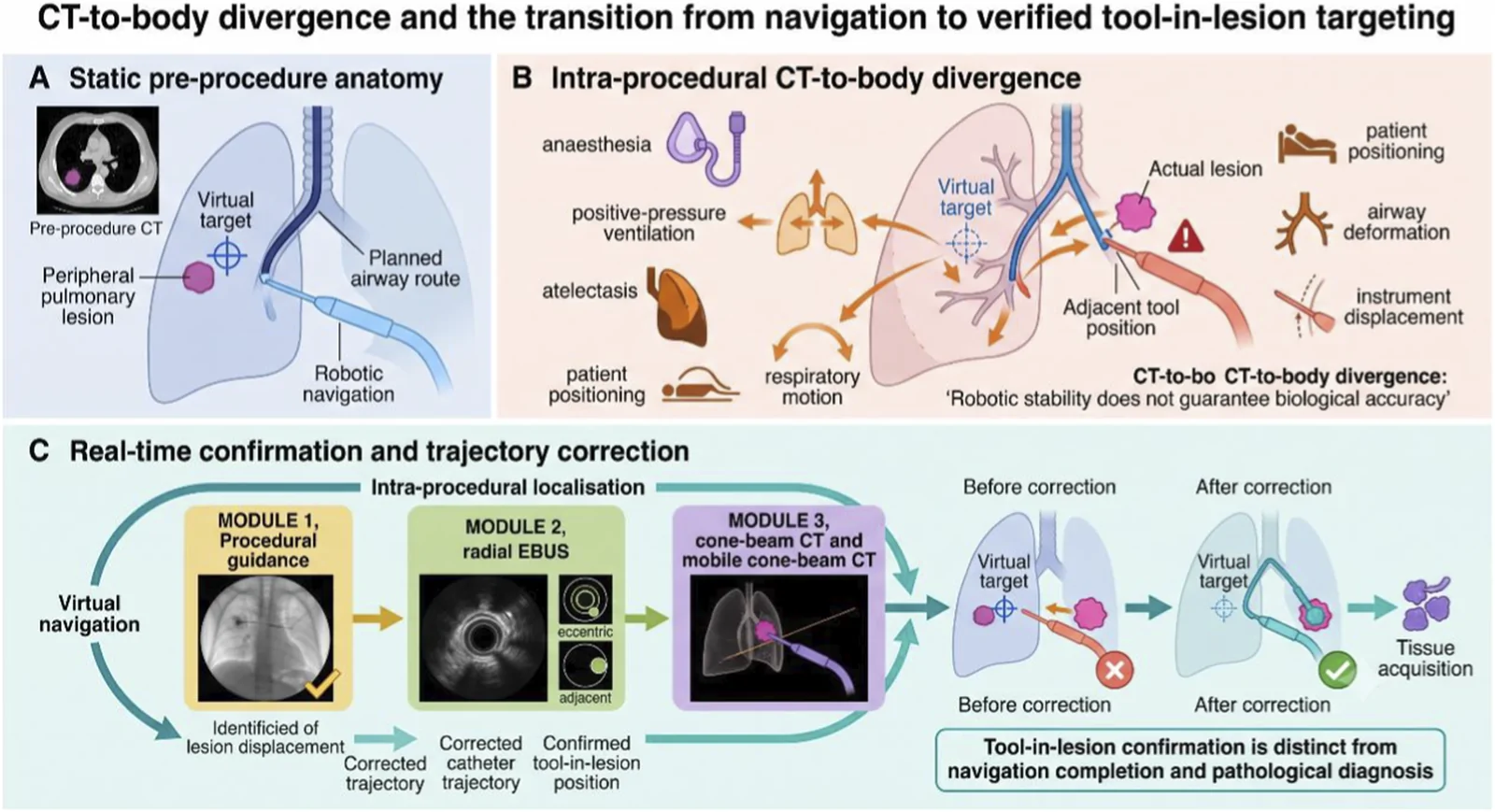
CT-to-body divergence and the transition from navigation to verified tool-in-lesion targeting. **(A)** Robotic navigation is initially based on static pre-procedural CT anatomy. **(B)** Anaesthesia, positive-pressure ventilation, atelectasis, respiratory motion, patient positioning and airway deformation can displace the actual lesion from the virtual target. **(C)** Intra-procedural imaging and radial endobronchial ultrasound allow localisation of the true target, trajectory correction and confirmation of tool-in-lesion position before tissue acquisition. Tool-in-lesion confirmation is therefore distinct from navigation completion and pathological diagnosis.

## 5 From diagnostic yield to clinically actionable yield

Diagnostic yield has been the dominant endpoint in studies of robotic bronchoscopy. Although it remains an essential measure of procedural performance, diagnostic yield is increasingly insufficient as a stand-alone marker of clinical value (Gonzalez et al., 2024; Vachani et al., 2023; Zhang et al., 2024). Its definition varies across studies, particularly in relation to non-specific benign findings, follow-up confirmation, repeat procedures, and adjudication of false-negative results. These inconsistencies limit comparison between studies and can overestimate the practical value of a procedure if diagnostic yield is interpreted without considering the quality and clinical utility of the obtained sample (Vachani et al., 2022; Sainz et al., 2024).

More importantly, diagnostic yield does not capture the full set of information required for contemporary lung cancer care. In suspected or confirmed non-small cell lung cancer, tissue is no longer used only to distinguish benign from malignant disease or to determine histological subtype. It is also expected to support immunohistochemistry, PD-L1 testing, broad molecular profiling, assessment of targetable alterations, and sometimes evaluation of resistance mechanisms (Connolly et al., 2023; Fox et al., 2024). In early-stage disease, the bronchoscopic procedure may also need to inform mediastinal staging, suitability for surgery or stereotactic body radiotherapy, eligibility for local ablation, or the need for surveillance (Fernandez-Bussy et al., 2025b; Dhaliwal et al., 2022). A procedure that establishes malignancy but fails to provide adequate tissue for molecular or staging assessment may therefore remain clinically incomplete.

We propose that robotic bronchoscopy can be evaluated through the concept of clinically actionable yield. This framework asks whether a bronchoscopic procedure not only reaches and diagnoses a lesion, but also generates sufficient information to support staging, molecular profiling, treatment selection, and longitudinal management. In this model, diagnostic yield is not discarded; rather, it becomes one component of a broader pathway that links procedural accuracy to clinical decision-making (Lee-Mateus et al., 2024; Fernandez-Bussy et al., 2025b). This framework is intended as a pragmatic reporting construct rather than a validated surrogate endpoint, and should be tested prospectively before it is used for comparative-effectiveness claims.

Clinically actionable yield can be conceptualised as a five-level hierarchy. The first level is lesion reached, reflecting the ability of the bronchoscopic platform to access the planned target region. The second is tool-in-lesion confirmed, ideally supported by real-time imaging, cone-beam CT, mobile cone-beam CT, or radial endobronchial ultrasound (Senyei et al., 2024; Murgu et al., 2025; Chan et al., 2026; Abia‐Trujillo et al., 2024). The third is pathologic diagnosis achieved, including cytological or histological confirmation. The fourth is molecular adequacy confirmed, indicating that the sample is sufficient for clinically relevant biomarker testing, such as next-generation sequencing and PD-L1 assessment where appropriate. The fifth level is treatment decision informed or changed, defined as a documented management decision that is directly informed or modified by information generated from the index bronchoscopic procedure. For prospective evaluation of this proposed framework, Level 5 should be assessed within 30 days after the index procedure or before definitive therapy, whichever occurs first. Each component of the hierarchy should be reported separately, and the composite should not be interpreted as a validated surrogate endpoint until prospectively evaluated.

This hierarchy is particularly important for robotic bronchoscopy because its technical promise lies not only in reaching peripheral pulmonary lesions, but in enabling a more complete procedural workflow. Stable catheter positioning and improved distal access may increase the likelihood of obtaining diagnostic material, but clinical value ultimately depends on how that material is processed, interpreted, and integrated into treatment planning (Paez et al., 2025a). Rapid on-site evaluation, cell block preparation, biopsy tool selection, cryobiopsy, same-session EBUS staging, and tissue stewardship are therefore not ancillary details; they are central determinants of whether robotic bronchoscopy delivers actionable information.

The shift from diagnostic yield to clinically actionable yield also has implications for research design. Future studies need to report not only diagnostic yield and complications, but also tool-in-lesion confirmation, CT-to-body divergence correction, tissue adequacy for molecular profiling, need for repeat biopsy, time to treatment, changes in multidisciplinary treatment decisions, and cost per actionable diagnosis (Chan et al., 2026). For studies evaluating bronchoscopic ablation, clinically actionable yield can be extended to include local control, pulmonary function preservation, quality of life, and comparison with established treatments such as surgery, stereotactic body radiotherapy, and percutaneous ablation.

Reframing robotic bronchoscopy in this way avoids both underestimating and overclaiming its value. It prevents the technology from being judged solely by narrow diagnostic endpoints, but it also requires that future claims of clinical benefit be linked to measurable decision-centred and patient-centred outcomes. Robotic bronchoscopy can therefore be assessed not only by whether it reaches the lesion, but by whether it improves the quality, completeness, and efficiency of early lung cancer care (Figure 3).

**FIGURE 3:**
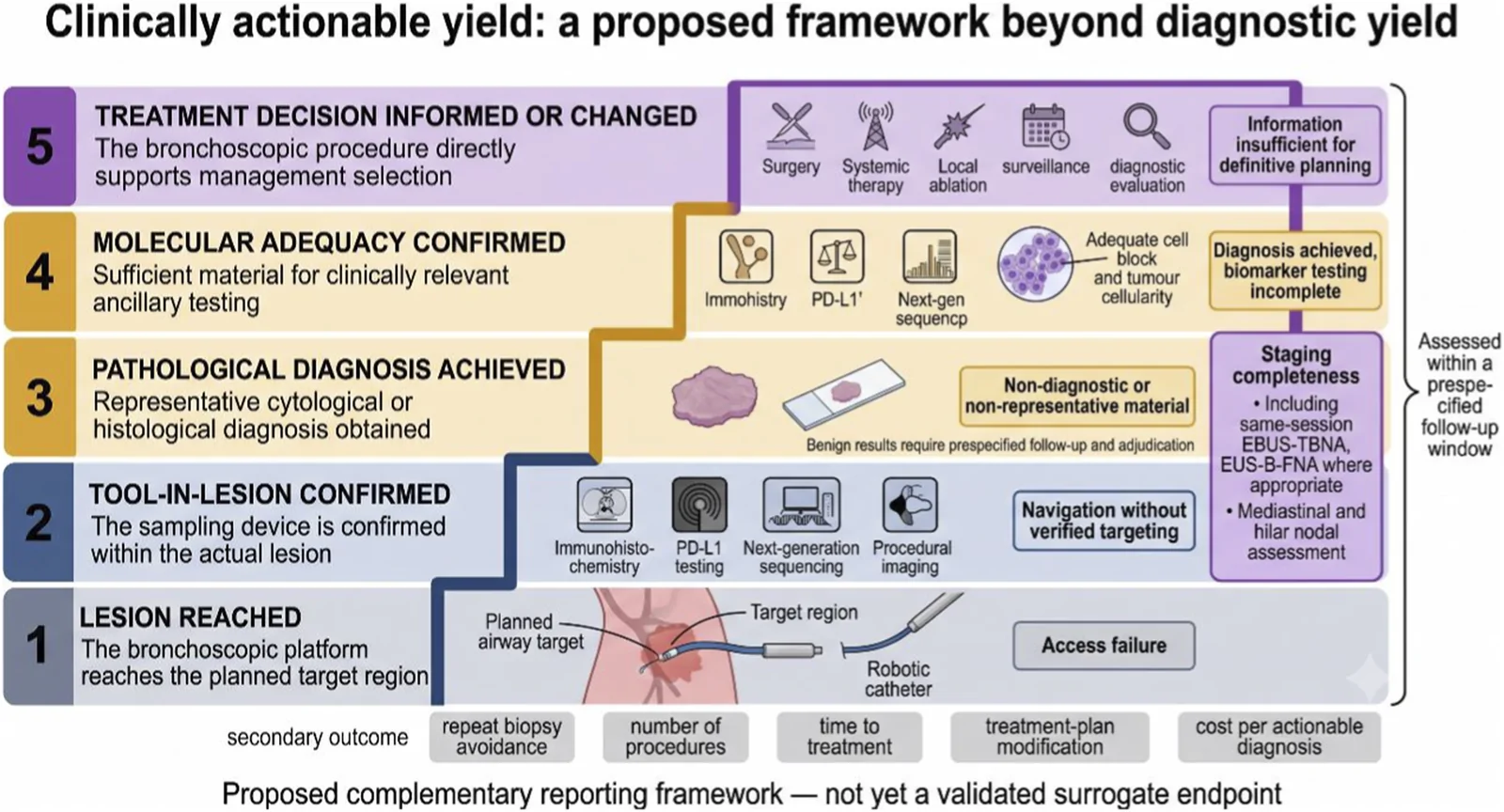
Proposed framework of clinically actionable yield in robotic bronchoscopy. Clinically actionable yield is conceptualised as a five-level hierarchy progressing from lesion reached, to tool-in-lesion confirmed, pathological diagnosis achieved, molecular adequacy confirmed, and ultimately treatment decision informed or changed. Staging completeness and downstream outcomes—including repeat-biopsy avoidance, number of procedures, time to treatment, treatment-plan modification and cost per actionable diagnosis—provide complementary measures of clinical value. The framework is proposed as a reporting construct and is not yet a validated surrogate endpoint. For prospective studies, Level 5 should be assessed within 30 days of the index bronchoscopic procedure or before definitive therapy, whichever occurs first.

## 6 Precision bronchoscopic biopsy, genomic profiling, and molecular adequacy

The clinical value of robotic bronchoscopy depends not only on reaching peripheral pulmonary lesions, but also on obtaining tissue that is adequate for modern thoracic oncology. In suspected non-small cell lung cancer, bronchoscopic specimens are expected to provide more than a binary benign or malignant diagnosis. They may need to support histological subtyping, immunohistochemistry, PD-L1 testing, next-generation sequencing, assessment of targetable alterations, and occasionally evaluation of resistance mechanisms (Chaddha et al., 2025; Kalchiem-Dekel et al., 2025). This requirement changes the procedural goal from diagnosis alone to tissue stewardship (Naso et al., 2023).

Tissue stewardship begins before the biopsy tool is deployed. Lesion characteristics, suspected disease stage, probability of malignancy, anticipated molecular-testing needs, and the possibility of same-session mediastinal staging can influence the sampling strategy (Chen et al., 2024). Small solid nodules, subsolid lesions, suspected adenocarcinoma, recurrent disease, and lesions in patients who may not tolerate repeat procedures require particular attention to sample adequacy. In these settings, the operator must consider not only which tool is most likely to establish a diagnosis, but which combination of tools is most likely to generate enough material for treatment selection (Diep et al., 2023).

Different bronchoscopic sampling methods provide different types of material. Forceps biopsy is widely available and familiar, but samples may be small or crushed. Needle aspiration can provide useful cytological material and cell block preparation, but its molecular utility depends on tumour cellularity and processing quality. Brushings and washings may supplement cellular yield, although their tumour fraction can be variable. Transbronchial cryobiopsy may provide larger and better-preserved tissue fragments, which could be advantageous for molecular profiling, but it requires attention to bleeding risk, tool compatibility, and procedural expertise (Meng et al., 2023; Husta et al., 2025). EBUS-TBNA remains essential when mediastinal or hilar nodal sampling is required, and EUS-B-FNA can extend access to selected nodal stations (Issa et al., 2024).

Rapid on-site evaluation can improve tissue stewardship by confirming diagnostic material during the procedure and guiding additional sampling when the initial material is inadequate (Roy‐Chowdhuri et al., 2024; Fowell and Khan, 2024). However, rapid on-site evaluation need not be viewed only as a tool for immediate diagnosis. In the era of precision oncology, it can also help determine whether sufficient material remains for ancillary testing. This requires coordination among the bronchoscopist, cytopathologist, molecular laboratory, and oncology team. Cell block preparation, slide triage, tumour cellularity assessment, and preservation of material for next-generation sequencing are practical determinants of whether a bronchoscopic sample becomes clinically actionable (Miller et al., 2025).

The concept of molecular adequacy is particularly important in early lung cancer care. Immune biomarker assessment represents a complementary dimension of tissue adequacy. PD-L1 remains an established tissue-based biomarker used in the clinical evaluation of immunotherapy strategies, although its interpretation is dependent on disease stage, treatment setting, assay methodology, and the broader molecular context. For bronchoscopic specimens, the practical requirement is therefore not simply to obtain a malignant diagnosis, but to preserve sufficient viable tumour material for reproducible immune-biomarker assessment when clinically indicated. This consideration further links biopsy technique and tissue stewardship to downstream immunotherapy-oriented decision-making. Although broad molecular testing is most established in advanced non-small cell lung cancer, tissue obtained during early diagnostic work-up may still influence treatment planning, trial eligibility, adjuvant or neoadjuvant strategies, and management of multifocal disease (Tsuboi et al., 2023). In addition, as systemic therapy moves earlier in the disease course, the boundary between diagnostic tissue and treatment-defining tissue is becoming less distinct. Robotic bronchoscopy can therefore be assessed not only by whether it diagnoses malignancy, but also by whether it preserves the option for genomically and immunologically informed treatment selection (Forde et al., 2022).

Same-session nodal staging further increases the value of bronchoscopic tissue acquisition. A single procedural session that combines robotic sampling of a peripheral lesion with EBUS-guided mediastinal staging can provide both diagnosis and stage-relevant information. This may reduce repeat procedures, shorten the interval from diagnosis to treatment, and improve multidisciplinary planning (Wong et al., 2024). However, the benefit of same-session staging depends on procedural sequencing, appropriate nodal selection, adequate sampling passes, and tissue processing that balances cytological diagnosis with molecular needs.

When bronchoscopic tissue is insufficient, complementary strategies may be needed. Liquid biopsy can provide a minimally invasive rescue pathway, particularly when repeat tissue sampling is unsafe or delayed, although sensitivity may be limited in early-stage disease (Mar et al., 2024). Targeted bronchial washing fluid and other bronchoscopic liquid-based samples are emerging as potential adjuncts, but their role requires further validation (Wu Y. et al., 2025). These approaches are best framed as complementary rather than substitutive until their diagnostic and molecular performance are established across lesion types and stages (Kim et al., 2026).

Future robotic bronchoscopy studies therefore need to report molecular-testing outcomes alongside diagnostic yield. Relevant endpoints include tumour cellularity, sample volume, cell block adequacy, PD-L1 success rate, next-generation sequencing success rate, need for repeat biopsy, time from procedure to molecular result, and whether molecular findings changed treatment (Wang et al., 2025). Without these data, it remains impossible to determine whether robotic bronchoscopy improves precision oncology or merely increases the rate of pathological diagnosis. From a genetics perspective, tissue adequacy should therefore be considered in relation to the ability of a specimen to preserve representative tumour DNA and RNA for clinically indicated genomic testing. The relevance of robotic bronchoscopy extends beyond obtaining sufficient tissue volume: tumour cellularity, nucleic-acid quality, specimen processing, and allocation of material across histology and molecular assays can all influence whether genomic profiling is successfully completed. This distinction is particularly important when small peripheral lesions provide limited tissue, because a diagnostically positive specimen may still be inadequate for comprehensive biomarker characterization.

## 7 Same-session staging and multidisciplinary treatment decision-making

The diagnosis-to-treatment value of robotic bronchoscopy is greatest when peripheral lesion sampling is integrated with staging and multidisciplinary decision-making. In suspected lung cancer, establishing malignancy is only one part of the clinical pathway. Treatment selection depends on tumour stage, nodal status, histological subtype, molecular profile, pulmonary reserve, comorbidities, lesion morphology, patient preference, and local expertise (Zer et al., 2025; Spicer et al., 2024). A bronchoscopic procedure that provides diagnosis but leaves staging incomplete may delay definitive management and require additional invasive procedures (Brunelli et al., 2025).

When clinically indicated, same-session mediastinal staging can be an important component of a bronchoscopic diagnosis-to-treatment platform. Robotic bronchoscopy can be used to sample a peripheral pulmonary lesion, while EBUS-TBNA and, where appropriate, EUS-B-FNA can be performed in the same procedural session to assess mediastinal and hilar lymph nodes (Korevaar et al., 2026). This integrated approach is particularly relevant when imaging suggests possible nodal involvement, when the primary lesion is suspicious for non-small cell lung cancer, or when treatment decisions would change according to nodal status (Oki et al., 2024). Combining peripheral lesion biopsy and nodal staging can reduce diagnostic fragmentation and shorten the interval between suspicion, diagnosis, staging, and treatment planning. However, routine same-session invasive mediastinal staging is not indicated for all peripheral pulmonary lesions. Patient selection should be guided by pre-procedural assessment of the likelihood of nodal metastasis and by whether nodal status is expected to alter management. A combined procedure may also prolong general anaesthesia and overall procedural time and increase cumulative procedural burden; these potential disadvantages should be balanced against the benefit of avoiding additional procedures (Palleiko et al., 2025).

The benefit of same-session staging also depends on appropriate procedural sequencing. When systematic nodal staging is indicated, sampling should follow oncological staging principles, including prioritisation of higher-stage nodal stations to minimise contamination-related false upstaging (Korevaar et al., 2026; Bradley et al., 2025). The relative sequence of nodal staging and peripheral lesion biopsy within a combined robotic procedure is less well standardised and may reasonably be individualised according to radiological suspicion, lesion accessibility, nodal findings, anaesthesia time, bleeding risk, and anticipated downstream tissue requirements (Gilbert et al., 2025).

This integrated workflow also changes the role of the bronchoscopist. The bronchoscopist is no longer only a proceduralist obtaining diagnostic tissue, but a contributor to the multidisciplinary treatment pathway. Pre-procedural planning can involve review of imaging, lesion morphology, bronchus sign, nodal stations, expected tissue requirements, pulmonary function, and potential treatment options. Communication with pathology and oncology teams before the procedure can help determine whether additional tissue can be preserved for next-generation sequencing, PD-L1 testing, clinical trial screening, or repeat molecular analysis. This biomarker-oriented workflow is particularly relevant as treatment selection increasingly integrates genomic alterations with immune biomarkers. In selected patients, the molecular profile of the tumour may support targeted-therapy strategies, whereas immune-biomarker information can contribute to decisions regarding perioperative or systemic immunotherapy. Robotic bronchoscopy may therefore function as an upstream tissue-acquisition component of precision oncology by linking technically successful lesion sampling with the genomic and immunological information required for downstream therapeutic stratification.

The clinical utility of same-session diagnosis and staging is most apparent in early lung cancer care. For operable patients, bronchoscopic diagnosis and nodal assessment may clarify whether surgery, sublobar resection, lobectomy, or additional mediastinal evaluation is appropriate. For medically inoperable patients, the same information may guide stereotactic body radiotherapy, percutaneous ablation, bronchoscopic ablation, or surveillance (Howington et al., 2025). For patients with multiple ground-glass or subsolid lesions, bronchoscopic information must be interpreted within a broader strategy that distinguishes dominant invasive lesions from indolent or multifocal disease (Wu H. et al., 2025).

Robotic bronchoscopy may also support treatment planning beyond tissue diagnosis. Accurate bronchoscopic localisation can inform surgical planning, lesion marking, selection of a target lesion among multiple nodules, or assessment of whether a lesion is suitable for a bronchoscopic therapeutic approach (Fernandez-Bussy et al., 2024b; Perez et al., 2025). However, such applications require careful evaluation because a technically reachable lesion is not necessarily an appropriate target for local therapy. Treatment suitability depends on lesion size, margin requirements, proximity to vessels and pleura, histology, growth pattern, pulmonary reserve, and competing treatment options (Gu et al., 2024). Single-anesthetic biopsy-to-resection workflows may be appropriate in carefully selected surgically fit patients when resection is already planned and when pre-operative pathological or molecular information is unlikely to alter the treatment strategy. Conversely, a staged approach may be preferable when molecular profiling could influence neoadjuvant treatment selection, when the biopsy result may change the indication or extent of surgery, or when additional multidisciplinary review is required before definitive treatment. Thus, procedural efficiency should not replace appropriate pre-operative oncological decision-making.

Future studies need to evaluate same-session workflows as clinical pathways rather than isolated procedures. Relevant endpoints include time from abnormal imaging to diagnosis, time from diagnosis to treatment, number of procedures per patient, proportion of patients receiving complete staging, molecular-testing success, multidisciplinary treatment-plan modification, patient-reported procedural burden, and cost-effectiveness (Zhang et al., 2022). These endpoints are better aligned with real-world care than diagnostic yield alone and would clarify whether robotic bronchoscopy improves the efficiency and completeness of early lung cancer management (Figure 4) (Weiser et al., 2025).

**FIGURE 4:**
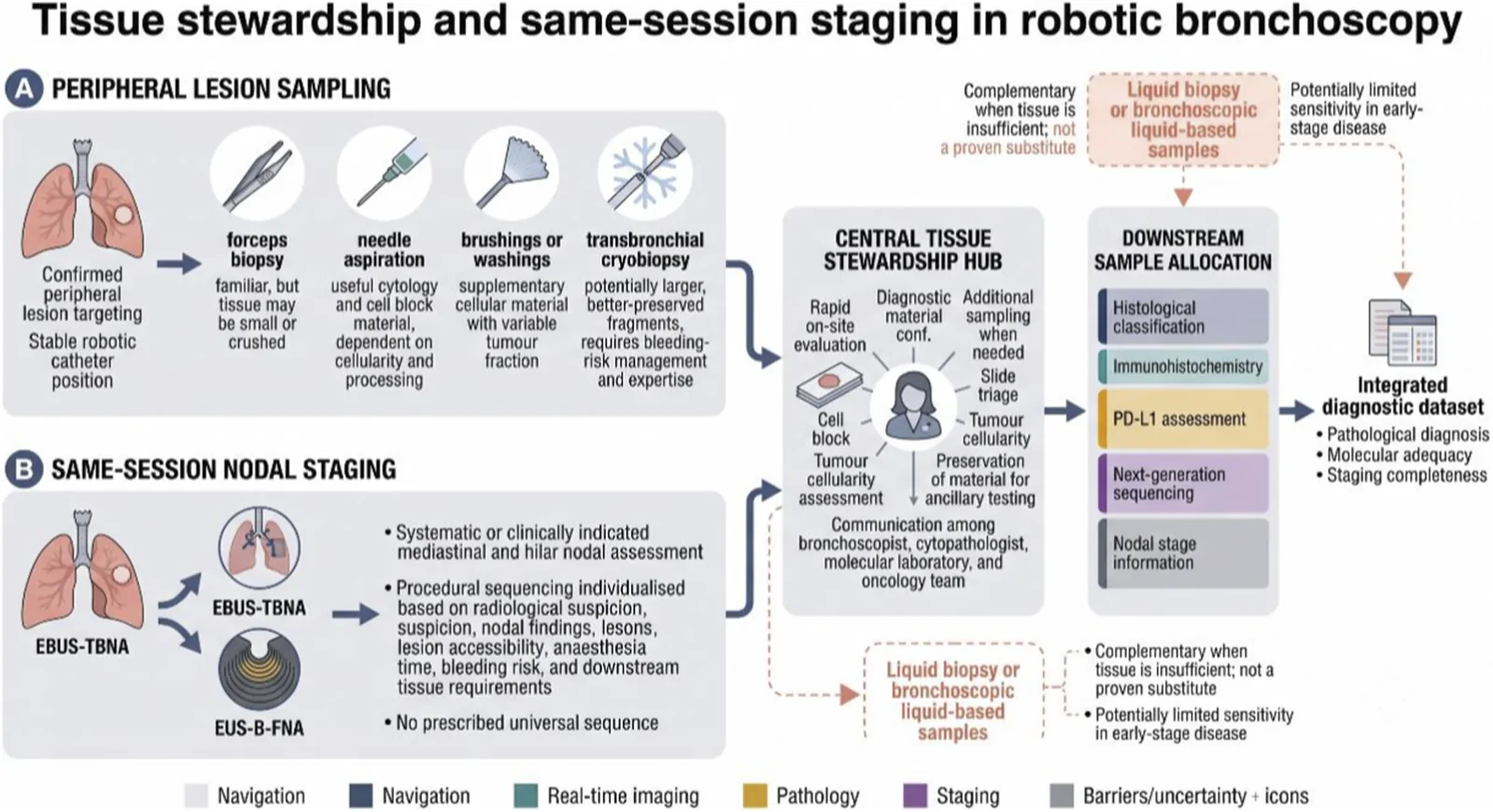
Tissue stewardship and same-session staging in robotic bronchoscopy. **(A)** Peripheral lesion sampling may combine forceps biopsy, needle aspiration, brushing or washing, and transbronchial cryobiopsy according to lesion and tissue requirements. **(B)** Same-session EBUS-TBNA and, when appropriate, EUS-B-FNA can provide complementary mediastinal and hilar nodal staging. Centralised tissue stewardship integrates rapid adequacy assessment, cell-block preparation, tumour cellularity assessment and specimen allocation for histology, immunohistochemistry, PD-L1 testing and next-generation sequencing. Liquid biopsy and bronchoscopic liquid-based samples are shown as complementary approaches when tissue is insufficient rather than established substitutes for tissue sampling.

## 8 Therapeutic extension: bronchoscopic ablation and local therapy integration

The therapeutic extension of robotic bronchoscopy is one of the most promising but least mature areas in interventional pulmonology. If robotic platforms can reliably reach peripheral pulmonary lesions, confirm tool-in-lesion position, and obtain diagnostic tissue, it is logical to ask whether the same access pathway can support local treatment. Bronchoscopic ablation represents this potential transition from diagnostic bronchoscopy to bronchoscopic interventional oncology (Xu et al., 2026; Fernandez-Bussy et al., 2026). Importantly, bronchoscopic ablation remains investigational. Accordingly, the technical prerequisites and clinical outcomes discussed below should be interpreted as candidate research considerations for future studies rather than validated treatment standards or established clinical indications.

The clinical rationale is clear. Low-dose CT screening and improved imaging have increased the detection of early-stage lung cancers, multiple ground-glass nodules, and small peripheral lesions in patients who are elderly, frail, or have limited pulmonary reserve (Adams et al., 2023). Some patients are medically inoperable, some are poor surgical candidates, and some decline surgery. For these patients, local treatment options such as stereotactic body radiotherapy and image-guided percutaneous ablation are already important alternatives (Pennathur et al., 2025). A bronchoscopic approach could theoretically offer a less pleural-disruptive route, reduce pneumothorax risk in selected patients, and enable diagnosis, staging, and treatment planning within a single procedural platform (Lau et al., 2023).

Several energy modalities are being explored for bronchoscopic local therapy, including microwave ablation, radiofrequency ablation, cryoablation, pulsed electric field ablation, and photodynamic therapy (Mahmood et al., 2026). Each modality has distinct biophysical properties, potential advantages, and technical limitations. Microwave ablation may generate larger and faster thermal zones; radiofrequency ablation has a longer history in local tumour treatment; cryoablation may offer tissue-preserving immunological effects and a different safety profile; pulsed electric field ablation may theoretically spare collagenous structures; and photodynamic therapy may be suitable for selected endobronchial or peribronchial lesions. However, the evidence base remains heterogeneous and early (Sun et al., 2024).

Bronchoscopic ablation is therefore best framed as an emerging extension rather than an established replacement for surgery, stereotactic body radiotherapy, or percutaneous ablation. Current early-phase evidence primarily supports technical feasibility and preliminary safety, while the evidence base remains limited by small sample sizes, short follow-up, heterogeneous lesion types, variation in ablation technology, inconsistent definitions of technical success, and limited long-term oncological endpoints (Zhong et al., 2025; Yang et al., 2026). Optimal ablation-margin definitions, durable local control, progression-free survival, overall survival, pulmonary function preservation, quality of life, retreatment rates, cost-effectiveness, and comparative effectiveness against established local therapies remain insufficiently defined or unproven. Without such data, it is premature to claim oncological equivalence with established local therapies.

Targeting reliability is the key prerequisite for bronchoscopic ablation. For diagnostic biopsy, small deviations in tool position may reduce yield or require repeat sampling. For ablation, the consequence of targeting error is more serious: incomplete tumour coverage, inadequate margins, collateral thermal injury, or false reassurance after treatment. For ablation, targeting error may result in incomplete tumour coverage or collateral injury; therefore, the lesion-confirmation principles described in Section 4 become even more stringent when the platform is extended from biopsy to therapy (Zhang et al., 2025; Hong et al., 2025).

Patient selection will determine whether bronchoscopic ablation becomes clinically meaningful. Potential candidates may include patients with medically inoperable early-stage non-small cell lung cancer, high surgical risk, poor pulmonary reserve, multiple lesions requiring staged management, or selected peripheral lesions where a bronchoscopic route is anatomically favourable. However, lesion size, solid versus subsolid morphology, distance from pleura, proximity to vessels and bronchi, histology, expected ablation margin, and availability of alternative treatments must all be considered (Chang et al., 2024). A lesion that is technically reachable by robotic bronchoscopy is not automatically an appropriate ablation target. Particular caution is required for ground-glass-dominant nodules, some of which may demonstrate indolent biological behaviour and may be appropriately managed with active surveillance. Technical accessibility alone should therefore not be considered an indication for bronchoscopic ablation.

Bronchoscopic ablation also raises workflow questions. Key unresolved issues include whether tissue diagnosis and ablation should occur in the same session or in staged procedures; whether molecular results should be available before local therapy; how local response should be assessed on post-ablation imaging; what constitutes complete ablation for subsolid lesions; and how residual or recurrent disease should be managed (Xu et al., 2025; Chang et al., 2024; Beattie et al., 2025). These questions are especially relevant in early-stage lung cancer, where overtreatment, undertreatment, and misclassification of indolent lesions all have consequences.

Future bronchoscopic ablation studies require designs that address these uncertainties directly. Early-phase studies can standardise technical success, targeting confirmation, ablation-zone assessment, complications, and short-term local control. Later trials can compare bronchoscopic ablation with stereotactic body radiotherapy, percutaneous ablation, or surgical approaches in carefully selected populations. Endpoints should include local control, progression-free survival, overall survival, pulmonary function, quality of life, need for retreatment, and health-economic outcomes. Importantly, studies should distinguish between the feasibility of delivering ablation bronchoscopically and the clinical value of doing so.

Bronchoscopic ablation is therefore best understood as a future-facing component of the robotic bronchoscopy platform. Its promise lies in the possibility of uniting lesion access, confirmation, diagnosis, staging, and local treatment planning (Xu et al., 2025). Its risk lies in overextension before adequate oncological evidence is available. A balanced research agenda needs to recognise both: robotic bronchoscopy may help create the technical conditions for bronchoscopic local therapy, but proof of durable clinical benefit remains the decisive requirement (Figure 5).

**FIGURE 5:**
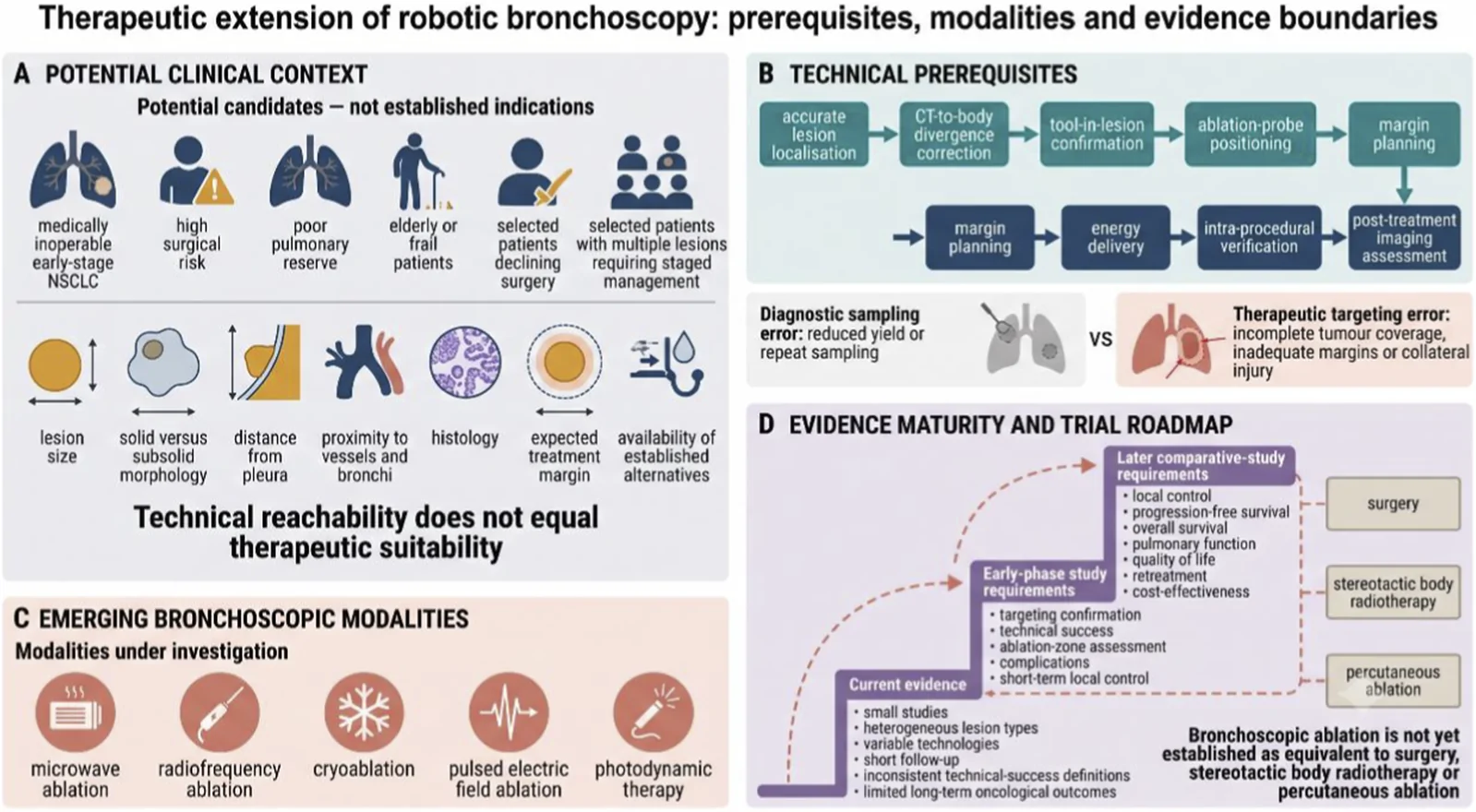
Therapeutic extension of robotic bronchoscopy: prerequisites, candidate modalities and evidence boundaries. Evidence boundary: bronchoscopic ablation remains investigational, and the variables shown in this figure represent candidate research prerequisites and endpoints rather than established clinical standards or validated indications. **(A)** Potential clinical contexts and lesion characteristics relevant to patient selection for bronchoscopic local therapy; these represent candidate scenarios rather than established indications. **(B)** Accurate localisation, correction of CT-to-body divergence, tool-in-lesion confirmation, treatment-margin planning and intra-procedural verification represent candidate technical prerequisites for future bronchoscopic ablation studies. **(C)** Emerging bronchoscopic modalities include microwave ablation, radiofrequency ablation, cryoablation, pulsed electric field ablation and photodynamic therapy. **(D)** Current evidence remains early, and progression from feasibility studies to comparative trials requires assessment of durable local control, survival, pulmonary function, quality of life, retreatment and cost-effectiveness against established local therapies.

## 9 Comparative clinical positioning of robotic bronchoscopy

Robotic bronchoscopy is best positioned against existing diagnostic pathways rather than evaluated in isolation. For pleural-based solid lesions in patients with acceptable pneumothorax risk, CT-guided transthoracic biopsy may remain efficient and can provide substantial tissue. For small, distal, bronchus-sign-positive lesions, especially when same-session staging or biomarker testing is anticipated, a bronchoscopic pathway may be more attractive (Nadig et al., 2023; Balasubramanian et al., 2024). For lesions without a bronchus sign, robotic stability alone cannot overcome absent airway access, and the relative advantage of transthoracic or alternative approaches may increase (Chan et al., 2026).

The recent randomized comparison of navigational bronchoscopy and transthoracic needle biopsy strengthens the rationale for including bronchoscopic routes in the work-up of technically amenable nodules, particularly because fewer pleural complications may matter in emphysema or limited pulmonary reserve. However, the choice among robotic bronchoscopy, conventional navigation, ultrathin bronchoscopy, radial EBUS, CT-guided biopsy, and combined workflows remain lesion- and patient-specific (Lentz et al., 2025; Vakil et al., 2025) (Table 1).

**TABLE 1:** Clinical positioning of robotic bronchoscopy within diagnostic pathways for peripheral pulmonary lesions.

Clinical context	Pathway most likely to be favoured	Rationale and key caveats	Refs.
Small, distal solid lesion with a favourable bronchus sign	Robotic or other advanced bronchoscopic biopsy	A navigable airway route, stable distal access and repeated tool deployment favour an endobronchial approach. Real-time confirmation remains important because navigation completion does not prove tool-in-lesion position	(Gonzalez et al., 2024; Leonard et al., 2024; Chan et al., 2026)
Small subsolid or ground-glass-dominant lesion	Individualised advanced bronchoscopic pathway with real-time three-dimensional confirmation	A bronchoscopic route may avoid pleural puncture and can support multi-site assessment, but low visibility and low tumour cellularity increase sampling error. Tool choice, trajectory correction and tissue preservation are critical	(Fernandez-Bussy et al., 2024a; Gomes et al., 2025; Abia-Trujillo et al., 2023)
Pleural-based solid lesion with a short, safe transthoracic path	CT-guided transthoracic needle biopsy	The transthoracic route may be efficient and can provide substantial tissue. Its relative advantage decreases when pneumothorax risk is high or when same-session nodal staging is required	(Lentz et al., 2025; Leonard et al., 2024)
No bronchus sign or no convincing navigable airway route	Transthoracic biopsy, surgical biopsy/resection or another alternative pathway	Robotic stability cannot compensate for absent airway access. Route selection should incorporate pleural distance, lesion morphology, operative candidacy and whether a tissue diagnosis would change management	(Gonzalez et al., 2024; Leonard et al., 2024)
Emphysema, limited pulmonary reserve or high anticipated pneumothorax risk	Bronchoscopic route when technically feasible	Avoiding transpleural puncture may be clinically valuable. However, anaesthesia, ventilation, atelectasis and procedure duration can still affect targeting and peri-procedural risk	(Lentz et al., 2025; Boster et al., 2026; Peralta and Debiane, 2026)
Need for mediastinal or hilar nodal staging	Robotic lesion sampling combined with same-session EBUS-TBNA and/or EUS-B-FNA	A single session can provide diagnosis and stage-relevant information. Nodal selection, procedural sequence and allocation of material between diagnosis and molecular testing should be planned in advance	(Lee-Mateus et al., 2024; Korevaar et al., 2026; Oki et al., 2024)
Need for broad biomarker testing, or repeat biopsy would be poorly tolerated	Integrated robotic workflow with multimodal sampling and formal tissue stewardship	Stable access permits complementary sampling tools and additional passes. Clinical value depends on tumour cellularity, cell-block preparation, rapid on-site feedback and preservation of material for PD-L1 and next-generation sequencing	(Fernande et al., 2026; Connolly et al., 2023; Kerr et al., 2024)
Multiple nodules or multifocal subsolid disease	Bronchoscopic pathway may be preferred for multi-target and nodal sampling	More than one lesion or nodal station may be assessed through a single airway-based session. The dominant lesion should be prioritised, and indolent lesions should not be overtreated solely because they are technically reachable	(Chaddha et al., 2025; Wu H. et al., 2025; Fernandez-Bussy et al., 2024b)
High pre-test probability of malignancy in a surgically fit patient when resection is already planned	Direct surgical diagnosis and treatment may be appropriate	An additional biopsy may be unnecessary when it will not alter the operative plan. The decision should be made in a multidisciplinary setting and should account for the likelihood of benign disease and the planned extent of resection	(Howington et al., 2025; Panel et al., 2023; Zer et al., 2025)
Medically inoperable patient being evaluated for local therapy	Bronchoscopic biopsy for diagnosis, staging and treatment planning; established local therapy selected separately	The airway platform may organise the diagnostic pathway, but bronchoscopic ablation remains investigational. Technical reachability should not be equated with therapeutic suitability or oncological equivalence	(Howington et al., 2025; Fernandez-Bussy et al., 2026; Pennathur et al., 2025)
Low-volume centre or limited access to advanced imaging and molecular pathology	The best-established local diagnostic pathway	Robotic bronchoscopy should be adopted only with an appropriate ecosystem of imaging, anaesthesia, pathology, training and quality assurance. The robot alone is unlikely to reproduce outcomes from expert centres	(Riestra Guiance and Kapp, 2025; Bertolaccini et al., 2025; Beyaz et al., 2025)

## 10 Implementation barriers: why technology alone is insufficient

The clinical adoption of robotic bronchoscopy is not best interpreted as a linear consequence of technical progress. Although robotic platforms may improve stability, reach, and procedural control, their real-world value depends on whether they can be implemented safely, consistently, and cost-effectively across diverse clinical settings (Riestra Guiance and Kapp, 2025). A technology that performs well in expert centres with advanced imaging, experienced teams, and mature pathology workflows may not produce the same outcomes in lower-volume centres or resource-limited environments (Bertolaccini et al., 2025).

Cost is one of the most important barriers. Robotic bronchoscopy requires major capital investment, disposable instruments, procedural space, anaesthesia support, imaging integration, technical staff, maintenance, and training. When cone-beam CT or mobile cone-beam CT is added, the resource requirement increases further. These costs may be justified if robotic bronchoscopy reduces complications, avoids repeat biopsies, shortens time to treatment, increases molecular-testing success, or enables more efficient staging (Bhandari and Ost, 2025). However, if its benefit is measured only by marginal improvements in diagnostic yield, the health-economic argument may remain uncertain (Bruinen et al., 2026).

The learning curve is another major determinant of implementation. Robotic bronchoscopy is not simply a new bronchoscope; it requires coordination among bronchoscopists, anaesthesiologists, radiographers, nurses, cytopathologists, molecular laboratories, and thoracic oncology teams (Bott et al., 2025). Operators must learn robotic navigation, imaging interpretation, CT-to-body divergence correction, tool deployment, bleeding management, sample triage, and procedural sequencing with EBUS staging. Institutions must also develop workflows for scheduling, equipment use, anaesthesia protocols, rapid on-site evaluation, and tissue stewardship (Liu et al., 2026). Without this multidisciplinary infrastructure, the platform may be underused or inconsistently applied.

Differences in anaesthesia and ventilation strategy may also influence outcomes. Positive-pressure ventilation, atelectasis, respiratory motion, airway deformation, and procedure duration can affect lesion position and CT-to-body divergence (Boster et al., 2026). These factors remain inconsistently standardised and reported across studies, yet they may substantially influence tool-in-lesion confirmation and diagnostic performance. Future protocols need to report ventilation strategy, use of recruitment manoeuvres, atelectasis prevention, airway pressure, patient positioning, and timing of intra-procedural imaging. Such details are not merely technical; they may explain why diagnostic yield differs across centres (Peralta and Debiane, 2026).

Endpoint heterogeneity remains a persistent problem. Diagnostic yield is variably defined, especially in relation to non-specific benign findings, follow-up duration, repeat procedures, and adjudication of false-negative results. Some studies report navigation success, others report lesion localisation, tool-in-lesion confirmation, pathological diagnosis, or sensitivity for malignancy. These endpoints are related but not interchangeable. Without standardised definitions, comparisons between platforms, imaging adjuncts, and sampling strategies are vulnerable to bias (Gonzalez et al., 2025).

Tissue adequacy is another underreported implementation issue. A procedure may be counted as diagnostically successful even if the sample is insufficient for next-generation sequencing, PD-L1 assessment, or clinical trial screening. For patients with suspected non-small cell lung cancer, this distinction matters. If molecular testing fails and repeat biopsy is required, the apparent diagnostic success may not translate into clinical efficiency. Studies therefore need to report tissue volume, tumour cellularity, cell block adequacy, molecular-testing success, need for repeat biopsy, and time from procedure to actionable result (Chaddha et al., 2025; Kanathanavanich et al., 2025).

Generalizability is also uncertain. Many encouraging data come from high-volume centres with expert operators and access to advanced imaging. This does not invalidate the findings, but it means that implementation research is needed. Pragmatic studies can include diverse institutions, varying levels of operator experience, and real-world patient populations. They can also evaluate how robotic bronchoscopy performs when integrated into routine diagnostic pathways rather than specialist research environments.

Therapeutic extension adds further implementation challenges. Bronchoscopic ablation requires an even higher level of targeting accuracy, procedural standardisation, complication management, and post-treatment imaging interpretation than biopsy. It also requires clear criteria for patient selection, ablation margins, response assessment, retreatment, and comparison with established local therapies. The threshold for evidence is therefore higher when robotic bronchoscopy is used for treatment rather than diagnosis.

Ultimately, the implementation question is not whether robotic bronchoscopy is technically impressive, but whether it improves care delivery. Its value is best judged by decision-centred and patient-centred outcomes: fewer repeat procedures, lower complication burden, higher molecular-testing success, shorter diagnostic intervals, more complete staging, better treatment selection, preserved lung function, improved quality of life, and acceptable cost-effectiveness. Without such evidence, technological sophistication alone will not be sufficient to justify broad adoption (Chan and Ng, 2025).

To improve feasibility across different resource settings, these implementation and patient-centred endpoints should be interpreted pragmatically. Core outcomes such as diagnostic performance, safety, repeat procedures, and time to treatment should be reported whenever possible, whereas detailed patient-reported and health-economic analyses may depend on local resources and validated data-collection systems. Cost per actionable diagnosis can be defined as the pathway-level cost divided by the number of patients achieving the prespecified actionable endpoint. Implementation barriers should be reported as contextual factors, including access to advanced imaging, anaesthesia support, rapid on-site evaluation, molecular pathology, trained personnel, and sufficient procedural volume.

## 11 Future research agenda

The next phase of robotic bronchoscopy research needs to move beyond whether the technology can reach peripheral pulmonary lesions and needs to ask whether it improves the completeness, efficiency, and quality of early lung cancer care. Future studies need to be designed around clinically meaningful endpoints rather than procedural enthusiasm (Brock et al., 2026; Roy et al., 2025).

First, the field needs standardised endpoint definitions. Diagnostic yield, navigation success, lesion localisation, tool-in-lesion confirmation, sensitivity for malignancy, and clinically actionable yield need to be clearly distinguished. Non-specific benign findings need to be handled consistently, with predefined follow-up requirements and transparent adjudication of false-negative results. A consensus reporting framework can allow more reliable comparison across robotic platforms, imaging adjuncts, sampling tools, and clinical settings (Gonzalez et al., 2024).

Second, prospective studies should quantify CT-to-body divergence using the technical variables defined in Section 4 and Table 2, including lesion displacement, atelectasis, ventilation strategy, imaging-based trajectory correction, and tool-in-lesion confirmation (Ho et al., 2025; Revelo et al., 2025).

**TABLE 2:** Proposed minimum reporting dataset for studies of robotic bronchoscopy.

Reporting domain	Core variables and operational definition	Recommended reporting and interpretation	Refs.
Patient and lesion profile	Age, comorbidity, pulmonary reserve and prior procedures; lesion size, density (solid, subsolid or ground-glass), lobe/segment, bronchus sign, distance from pleura, airway generation, multiplicity and pre-test probability of malignancy	Report for the full enrolled cohort and for the analysed cohort. Stratify key outcomes by lesion size, density and bronchus sign whenever sample size permits	(Miller et al., 2025; Brock et al., 2026)
Procedural environment	Robotic platform, imaging adjuncts, anaesthesia technique, ventilation strategy, patient position, atelectasis-prevention measures, operator experience, centre volume and availability of rapid on-site evaluation	Describe sufficiently to allow replication and to distinguish device performance from the performance of the wider procedural ecosystem	(Brock et al., 2026; Riestra Guiance and Kapp, 2025; Peralta and Debiane, 2026)
Navigation and lesion access	Navigation completion should mean that the catheter reached the planned target region. It should not be used as a synonym for lesion localisation, tool-in-lesion confirmation or diagnostic success	Report the proportion of all attempted lesions in which navigation was completed, with reasons for access failure	(Miller et al., 2025; Chan et al., 2026)
CT-to-body divergence	Presence and, where feasible, magnitude/direction of lesion displacement relative to the pre-procedure plan; associated atelectasis or deformation; whether intra-procedural imaging changed the catheter or tool trajectory	Report before sampling. Separate procedures requiring correction from those in which planned and actual target locations were concordant	(Ho et al., 2025; Revelo et al., 2025)
Tool-in-lesion confirmation	Confirmation modality (cone-beam CT, mobile cone-beam CT, radial EBUS, fluoroscopy or combinations), timing, and result. When radial EBUS is used, report concentric, eccentric or adjacent views; when three-dimensional imaging is used, report direct tool-in-lesion status	Use all attempted lesions as the denominator and report confirmation before the first diagnostic sample and after any trajectory correction	(Chan et al., 2026; Bhadra et al., 2023b; Revelo et al., 2025)
Sampling strategy	Sampling tools, sequence, number of passes, needle gauge, forceps/cryobiopsy/brushings/washings, suction technique, complications that limited sampling and use of rapid on-site evaluation	Report per lesion and identify which specimen type established the diagnosis and which supplied ancillary testing material	(Fernande et al., 2026; Chen et al., 2025; Meng et al., 2023)
Pathological diagnosis	Predefined diagnostic categories, including malignant, specific benign, non-specific benign and non-diagnostic results. Non-specific benign findings require a prespecified verification strategy	Report diagnostic yield and sensitivity for malignancy separately. State the follow-up duration, repeat procedures and adjudication rules for false-negative or indeterminate results	(Miller et al., 2025; Fernandez-Bussy et al., 2025a; Sainz et al., 2024)
Tissue and molecular adequacy	Tumour cellularity, tissue volume or fragment number where available, cell-block adequacy, immunohistochemistry success, PD-L1 success, next-generation sequencing success, assay type and laboratory failure reason	Use the number of patients for whom each test was clinically indicated as the principal denominator. Report turnaround time, repeat-biopsy requirement and whether biomarker results changed treatment	(Fernande et al., 2026; Connolly et al., 2023; Kerr et al., 2024)
Staging completeness	Whether EBUS-TBNA and/or EUS-B-FNA was performed; nodal stations sampled; adequacy of nodal material; final clinical/pathological stage; whether staging was completed in the same session	Report among patients for whom invasive staging was clinically indicated, rather than across the entire biopsy cohort	(Lee-Mateus et al., 2024; Korevaar et al., 2026; Oki et al., 2024)
Clinically actionable yield	A prespecified composite indicating that the procedure generated sufficient information for a management decision, incorporating pathological diagnosis and, when indicated, molecular adequacy and staging completeness	Specify the follow-up window and report every component separately. The composite should be treated as a pragmatic, prospectively testable construct rather than a validated surrogate endpoint	(Miller et al., 2025; Fox et al., 2024; Fernandez-Bussy et al., 2025b)
Safety	Pneumothorax, bleeding, hypoxaemia, respiratory failure, unplanned escalation of care, hospital admission, 30-day mortality and procedure-related events attributable to anaesthesia or adjunct imaging	Use standard severity grading and report immediate, discharge and 30-day outcomes. Distinguish events caused by biopsy, staging and therapeutic components	(Lentz et al., 2025; Senyei et al., 2024; Ali et al., 2023)
Efficiency and patient-centred outcomes	Number of procedures per patient, repeat biopsy, time from abnormal imaging to diagnosis, time from diagnosis to treatment, procedure duration, patient-reported burden, pulmonary function and quality of life where relevant	Use prespecified time points and compare with the alternative diagnostic pathway rather than reporting isolated procedural times only	(Miller et al., 2025; Zhang et al., 2022; Weiser et al., 2025)
Implementation and health economics	Capital and disposable costs, anaesthesia and imaging resource use, room occupancy, staffing, learning curve, case volume, cancellations, downstream procedures and cost per actionable diagnosis	Report from a defined health-system perspective and include sensitivity analyses for centre volume, complication avoidance and repeat-biopsy reduction	(Riestra Guiance and Kapp, 2025; Bhandari and Ost, 2025; Beyaz et al., 2025)
Future therapeutic-extension studies	Candidate variables for future trials include targeting confirmation, planned ablation margin, ablation-probe position, energy delivery, ablation-zone assessment, technical success, complications, local control, retreatment, pulmonary function, quality of life, progression-free survival and overall survival	Distinguish technical feasibility from durable oncological benefit. Comparative studies should use established treatments - surgery, stereotactic body radiotherapy or percutaneous ablation - as clinically appropriate comparators	(Fernandez-Bussy et al., 2026; Zhong et al., 2025; Beattie et al., 2025)

The proposed dataset should be adapted to local resources; not all secondary patient-reported or health-economic variables are expected to be available in every study.

Third, robotic bronchoscopy needs comparison against established diagnostic pathways. Randomized or pragmatic comparative studies need to evaluate robotic bronchoscopy against CT-guided transthoracic biopsy, electromagnetic navigation bronchoscopy, ultrathin bronchoscopy, or combined approaches in defined patient groups. Relevant endpoints should include clinically actionable yield, complications, need for repeat biopsy, time to treatment, patient-reported burden, and cost per actionable diagnosis. Such trials would be more informative than diagnostic-yield comparisons alone (Lentz et al., 2025; Paez et al., 2025b).

Fourth, studies need to evaluate molecular adequacy as a primary or key secondary endpoint. Future research needs to report tumour cellularity, sample volume, cell block adequacy, PD-L1 success, next-generation sequencing success, turnaround time, and treatment changes resulting from molecular findings. This is particularly important as systemic therapies and biomarker-driven strategies move into earlier stages of non-small cell lung cancer care (Zer et al., 2025).

Fifth, the value of same-session workflows need to be tested. Robotic sampling of peripheral lesions combined with EBUS or EUS-B staging may reduce diagnostic fragmentation, but this assumption requires evidence. Pragmatic studies need to measure the number of procedures per patient, completeness of staging, interval from abnormal imaging to diagnosis, interval from diagnosis to treatment, multidisciplinary treatment-plan modification, and healthcare utilisation (Ter Woerds et al., 2025; Styrvoky et al., 2024).

Sixth, bronchoscopic ablation requires a dedicated trial agenda. Early-phase studies need to standardise technical success, targeting confirmation, ablation-zone assessment, complications, and short-term local control. Later comparative studies need to evaluate bronchoscopic ablation against stereotactic body radiotherapy, percutaneous ablation, or surgery in carefully selected patients. Endpoints include local control, progression-free survival, overall survival, pulmonary function preservation, quality of life, retreatment, and cost-effectiveness. It will be essential to distinguish technical feasibility from oncological value (Fernandez-Bussy et al., 2026; Beattie et al., 2025).

Seventh, implementation science can be incorporated into robotic bronchoscopy research. Studies should evaluate learning curves, operator experience, centre volume, training requirements, workflow design, anaesthesia protocols, pathology coordination, and resource utilisation. These factors will determine whether robotic bronchoscopy can be scaled beyond expert centres (Brock et al., 2025; Beyaz et al., 2025) (Table 2).

Finally, future research should maintain a balanced standard of evidence. Robotic bronchoscopy need not be dismissed because it is expensive and technologically complex, nor accepted simply because it is innovative. Its long-term role can be determined by whether it improves diagnostic completeness, treatment readiness, patient experience, and outcomes compared with existing pathways.

## 12 Conclusion

Robotic bronchoscopy has matured from an emerging navigation technology into a potential procedural platform for peripheral pulmonary lesions. Its value lies not only in improved access to small and distal targets, but in the possibility of integrating lesion confirmation, tissue acquisition, molecular profiling, mediastinal staging, treatment planning, and future local therapy within a more coherent pathway for early lung cancer care.

Within this framework, genomic and immune biomarker adequacy represent increasingly important components of treatment readiness. A successful robotic bronchoscopic procedure should therefore be evaluated not only by pathological diagnosis but also, when clinically indicated, by its ability to provide sufficient material for genomic profiling and immune-biomarker assessment. This biomarker-driven perspective may help connect peripheral lesion sampling with precision targeted therapy, immunotherapy-oriented decision-making, and future molecularly stratified clinical trials. The field now needs to move beyond diagnostic yield as the dominant measure of success. Diagnostic yield remains necessary, but it is insufficient to capture whether a bronchoscopic procedure provides information that changes clinical management. We propose clinically actionable yield as a broader, currently unvalidated reporting framework linking lesion access, tool-in-lesion confirmation, pathological diagnosis, molecular adequacy, and treatment decision-making.

This broader framework also imposes a higher evidentiary standard. Robotic bronchoscopy can be assessed within the full ecosystem required for modern thoracic oncology, including real-time imaging, CT-to-body divergence correction, radial endobronchial ultrasound, tissue stewardship, rapid on-site evaluation, cell block preparation, molecular testing, EBUS staging, and multidisciplinary decision-making. The robot alone is not the platform; the integrated workflow is the platform.

Bronchoscopic ablation and other therapeutic extensions represent an important future direction, but current evidence remains early and heterogeneous. These approaches need to be developed through rigorous trials that evaluate targeting accuracy, local control, pulmonary function, quality of life, survival outcomes, retreatment, and cost-effectiveness. They must not be presented as established alternatives to surgery, stereotactic body radiotherapy, or percutaneous ablation until durable oncological evidence is available.

The next-generation of robotic bronchoscopy research needs to ask a more clinically relevant question: not simply whether the bronchoscope can reach the lesion, but whether the procedure improves the quality, completeness, efficiency, and outcomes of early lung cancer care.
